# Mushroom-Derived Hydrophobins for Antifouling and Interface Preservation in Chemosensors

**DOI:** 10.3390/s26051642

**Published:** 2026-03-05

**Authors:** Nardos F. Bisrat, Bethany R. Finnefrock, Matthew D. Gacura, Longyan Chen, Davide Piovesan

**Affiliations:** 1 Biomedical, Industrial and Systems Engineering Department, Gannon University, 109 University Square, Erie, PA 16541, USA; bisrat001@gannon.edu (N.F.B.); finnefro002@gannon.edu (B.R.F.); chen084@gannon.edu (L.C.); 2 Biology Department, Gannon University, 109 University Square, Erie, PA 16541, USA; gacura001@gannon.edu

**Keywords:** hydrophobins, antifouling coatings, anti-wetting surfaces, chemosensors, biosensors, medical device surfaces, surface modification, bio-based materials, tribology

## Abstract

Surface fouling remains a critical challenge for medical devices and chemosensor systems operating in biological environments, where nonspecific adsorption of proteins, cells, and microorganisms can lead to signal drift, reduced sensitivity, and shortened device lifetime. Conventional antifouling strategies rely primarily on synthetic hydrophilic polymer coatings, such as polyethylene glycol and polyvinylpyrrolidone, which are effective but face limitations related to long-term stability, thickness, and compatibility with surface-sensitive sensing modalities. In this review, we focus on hydrophobins derived from mushroom-forming and filamentous fungi as a bio-based alternative for antifouling and anti-wetting surface modification. Mushroom-derived hydrophobins are small amphiphilic proteins capable of spontaneous self-assembly into nanometer-scale films that modulate surface energy, wettability, and interfacial friction without requiring covalent functionalization. The current state of research on hydrophobin structure, classification, and self-assembly is reviewed, followed by a synthesis of reported antifouling and tribological behaviors relevant to medical and sensor-adjacent surfaces. Representative experimental observations are discussed to illustrate trends consistent with the literature, without establishing new performance benchmarks. The implications of mushroom-derived hydrophobin coatings for chemosensors and biosensors are examined, particularly with respect to signal stability, surface accessibility, and durability. Limitations and future research directions are outlined to support translation into practical sensing technologies.

## 1. Introduction

Surface fouling remains a fundamental limitation for medical devices and chemosensor systems operating in biological environments. Nonspecific protein adsorption, bacterial adhesion, and platelet accumulation at solid–liquid interfaces can lead to thrombosis, infection, signal drift, and reduced device lifetime, particularly in blood-contacting or biofluid-exposed applications [1,2,3]. For chemosensors and biosensors, even minor surface contamination can significantly degrade sensitivity, selectivity, and long-term stability, making antifouling surface engineering a critical enabling technology rather than a secondary design consideration [3,4].

Current industrial solutions rely predominantly on synthetic hydrophilic polymer coatings, most notably polyethylene glycol (PEG) and polyvinylpyrrolidone (PVP), which reduce fouling through hydration-layer formation and enhanced lubricity at the solid–liquid interface [5,6,7]. While effective in the short term, these coatings present well-documented limitations, including oxidative degradation, polymer chain leaching, sensitivity to sterilization processes, and emerging concerns related to immunogenic responses—particularly in the case of PEG [8,9,10].

Additional challenges arise in sensor-adjacent applications, where coating thickness and polymer chain mobility can interfere with analyte transport, interfacial mass transfer, and surface-sensitive signal transduction mechanisms, thereby impacting sensor sensitivity and response kinetics [3,11,12]. These limitations have motivated growing interest in alternative antifouling strategies that combine durability, biocompatibility, and minimal impact on sensor performance.

Hydrophobins, a class of small amphiphilic proteins produced by filamentous fungi, have emerged as promising bio-derived candidates for surface modification due to their unique interfacial activity and biological origin [2,4,13]. Their ability to spontaneously self-assemble into nanometer-thin films at hydrophilic–hydrophobic interfaces enables efficient modulation of surface energy, wettability, and interfacial friction without the thick polymer layers associated with conventional coatings. This self-assembly behavior, driven primarily by interfacial energy minimization rather than covalent bonding, has been widely documented on polymeric, metallic, and inorganic substrates relevant to biomedical devices and sensing applications [14,15,16].

However, divergent perspectives remain regarding the long-term stability, immunogenicity, and scalability of hydrophobin coatings, particularly when comparing Class I hydrophobins, which form mechanically robust amyloid-like rodlet structures, to Class II hydrophobins, which yield more reversible but less durable films [1,17,18]. These trade-offs are especially relevant for sensor-adjacent applications, where coating thickness, reversibility, and surface accessibility must be carefully balanced to preserve analyte transport and signal fidelity.

Importantly, the vast majority of hydrophobins investigated for antifouling and surface engineering applications are derived from filamentous fungi and mushroom-forming species, which represent the most practical and scalable biological sources of these proteins [19,20]. Mushroom-derived hydrophobins exhibit species-dependent variations in amino acid sequence, self-assembly behavior, and film organization, factors that can influence wettability modulation, antifouling efficacy, and tribological response [21,22,23]. Despite their widespread experimental use, these fungal-source-specific characteristics are often treated implicitly in the literature rather than explicitly considered when comparing coating performance or assessing suitability for device and sensor integration.

The aim of this review is therefore to critically synthesize current knowledge on mushroom-derived hydrophobin-based antifouling and anti-wetting films, in comparison with established PEG- and PVP-based systems, with specific emphasis on implications for medical devices and chemosensor interfaces [3,4]. By integrating mechanistic understanding, representative experimental observations, and application-driven considerations, this work highlights both the potential and the limitations of mushroom-derived hydrophobin coatings and outlines key directions for future research and translation.

### Scope and Novelty

While hydrophobins have been broadly discussed in prior reviews covering fungal biosurfactants and protein self-assembly [4,24,25,26], focused syntheses addressing mushroom-derived hydrophobins and their specific implications for chemosensor and biosensor interfaces remain comparatively limited, particularly for commonly cultivated macrofungal species used in biomaterials research. The present work, therefore, adopts a sensor-interface-centered perspective, emphasizing the relationships between antifouling behavior, tribological performance, surface accessibility, and signal stability in sensing platforms. In addition, the review integrates representative pilot experimental observations designed to illustrate literature-reported trends in controlled sensor-adjacent configurations, providing a practical bridge between fundamental interfacial science and application-oriented interpretation. These features position the manuscript as a mechanistically focused and application-driven synthesis rather than a purely descriptive survey.

## 2. Antifouling Challenges in Medical and Sensor-Adjacent Surfaces

Medical devices and chemosensor platforms that operate at solid–liquid interfaces face stringent—and often competing—surface-performance requirements. In biological environments, fouling processes such as protein adsorption, bacterial colonization, and platelet activation occur rapidly and can fundamentally alter interfacial properties, leading to impaired device function and reduced operational lifetime [9,27,28]. For blood-contacting devices, these effects can manifest as thrombosis or infection; for chemosensors and diagnostic probes, they more commonly appear as baseline drift, increased noise, sluggish response times, and reduced analytical accuracy due to partial obstruction of mass transport and changes in near-surface chemistry [3,29]. This creates a practical sensor interface paradox: sensors must remain sufficiently exposed to the environment to function, yet this exposure accelerates the very interfacial degradation mechanisms that compromise measurement fidelity [8,30].

Fouling is rarely a single event; it is typically a cascade. Within seconds to minutes, nonspecific adsorption of proteins and other macromolecules forms a conditioning layer that modifies surface energy and promotes subsequent biological attachment [27,31]. This conditioning film can facilitate bacterial adhesion and biofilm maturation, introducing mechanically robust and chemically heterogeneous layers that are difficult to remove without damaging the underlying transducer [9,28]. In parallel, blood-contacting systems face the additional complication that adsorbed proteins such as fibrinogen promote platelet adhesion and activation, directly linking surface fouling to thrombogenic risk [27,32]. Even when catastrophic failure does not occur, these time-dependent interfacial changes cause gradual sensor performance deterioration that cannot be corrected by signal processing alone, as the failure mode is physicochemical rather than purely electronic [3,30].

From an engineering perspective, effective antifouling surfaces must satisfy multiple criteria simultaneously. Coatings must adhere strongly to diverse substrates—including polymers, metals, and ceramics—while remaining stable under continuous hydration, mechanical stress, and clinically relevant sterilization or disinfection workflows [33]. At the same time, they must reduce nonspecific biological adhesion without introducing excessive thickness, pore blockage, or diffusion barriers—an especially critical constraint for chemosensors and biosensors, where signal transduction depends on near-surface interactions such as electron transfer, optical near-field coupling, or analyte diffusion to reactive sites [3,29]. In practice, coatings that perform well on medical device housings may fail on sensor-adjacent interfaces because the tolerance for added thickness and altered permeability is far smaller for sensing elements than for structural components [8,11].

Tribological performance represents an additional and frequently underappreciated challenge for sensor-adjacent surfaces. Many biomedical devices and sensor housings experience repeated mechanical contact with tissue, insertion tools, or flow-driven interactions, making low interfacial friction essential to minimize wear, tissue irritation, and coating delamination [10,34]. Hydrophilic polymer coatings are widely adopted to address lubricity and short-term fouling resistance; however, their reliance on hydrated polymer brush layers introduces vulnerabilities related to dehydration, oxidative degradation, chain scission, and long-term mechanical instability—particularly under repeated shear, flexure, or abrasion [7,35]. For sensor deployments requiring stable calibration over time, these degradation pathways translate directly into drift, reduced reproducibility, and loss of analytical confidence [3].

These constraints motivate increased interest in bio-derived antifouling strategies, including fungal hydrophobins, which rely on the formation of nanometer-scale, self-assembled films rather than thicker polymer brush layers [2,4,13]. For mushroom-derived hydrophobins in particular, the motivation is not only functional but also practical: edible and commercially cultivated fungi provide accessible biomass and may enable lower-barrier extraction routes compared with fully recombinant protein production, supporting early-stage prototyping and translational research [19,21,36]. At the same time, mushroom-derived preparations introduce additional engineering considerations, including batch-to-batch variability, co-extracted biomolecules, and species-dependent hydrophobin composition, all of which can influence film uniformity, wettability control, friction behavior, and sensor repeatability [22,23,37]. These factors are particularly important for chemosensor interfaces, where subtle changes in surface chemistry can lead to measurable differences in sensitivity and long-term drift [3,29].

A key point of debate, therefore, concerns the trade-off between coating robustness and functional compatibility with sensing. Highly stable coatings may provide long-term antifouling protection but risk irreversibility, difficult regeneration, or reduced analyte access to the sensing region, whereas more dynamic or reversible films may preserve surface functionality at the expense of durability [4,17]. In this context, hydrophobin-based coatings—particularly those sourced from mushrooms—should be evaluated not only on antifouling performance, but also on how they balance (i) film thickness and permeability, (ii) mechanical stability under shear and handling, (iii) compatibility with sterilization and cleaning protocols, and (iv) reproducibility across coating batches and fungal species [20,38]. These competing design priorities underscore the need for antifouling strategies that simultaneously address biocompatibility, tribological resilience, and surface sensitivity in sensor-adjacent and diagnostic applications.

## 3. Conventional Polymer-Based Antifouling Coatings: Performance and Limitations

Synthetic hydrophilic polymer coatings constitute the prevailing standard for antifouling and lubricious surface modification in medical devices and sensor-related technologies. Polyethylene glycol (PEG) and polyvinylpyrrolidone (PVP) are among the most widely adopted materials due to their established biocompatibility, processing versatility, and demonstrated effectiveness in reducing nonspecific protein adsorption and cellular adhesion [5,6,8]. Their antifouling performance arises primarily from the formation of hydrated surface layers that impose energetic and steric barriers at solid–liquid interfaces [12,28].

In many implementations, these polymers are deployed as “polymer brush” coatings, in which flexible chains are densely grafted to a substrate and tethered by one end, forcing them to extend into the surrounding aqueous environment. High grafting densities produce hydrated layers that can reach tens of nanometers in thickness and resist fouling through steric and entropic repulsion, as compression of the chains upon biomolecular approach is energetically unfavorable. While polymer brushes are effective antifouling architectures, their thickness, chain mobility, and hydration dynamics distinguish them structurally and functionally from ultrathin, self-assembled coatings such as hydrophobin films [39,40].

PVP-based coatings are commonly applied to catheters, guidewires, implants, and diagnostic probes, where rapid hydration and strong surface adhesion are required [5]. Their amphiphilic nature enables good wettability and low interfacial friction under aqueous conditions, which is particularly beneficial for devices experiencing mechanical insertion or sliding contact. However, PVP coatings are susceptible to leaching, mechanical wear, and gradual loss of lubricity during prolonged use, especially under repeated hydration–dehydration cycles or sustained shear [7,35]. These degradation pathways can compromise long-term antifouling performance, leading to progressive fouling and reduced device reliability in extended deployments [9].

PEG-based coatings are similarly valued for their strong resistance to protein adsorption and platelet activation, attributes that have supported their widespread use in blood-contacting devices and biosensors [8]. PEG chains form flexible hydration layers that effectively repel biomolecules through entropic and enthalpic penalties associated with dehydration [11]. Despite these advantages, PEG-based systems are vulnerable to oxidative degradation, chemical instability during sterilization, and chain scission under physiological conditions [12,41]. In addition, growing evidence of anti-PEG antibody formation has raised concerns regarding immunogenic responses and long-term clinical compatibility, particularly for implantable or repeatedly exposed devices [2].

From a chemosensor perspective, an additional limitation of polymer-based antifouling systems lies in their physical thickness and structural dynamics. Polymer brush layers—while effective fouling barriers—can interfere with surface-sensitive detection mechanisms by altering local dielectric properties, hindering analyte transport, or partially screening electrochemical or optical interactions occurring within the near-surface region [3,29]. Over time, polymer rearrangement, compression, or partial delamination can further contribute to signal attenuation and baseline drift, complicating calibration and long-term data interpretation [30].

**Figure 1:**
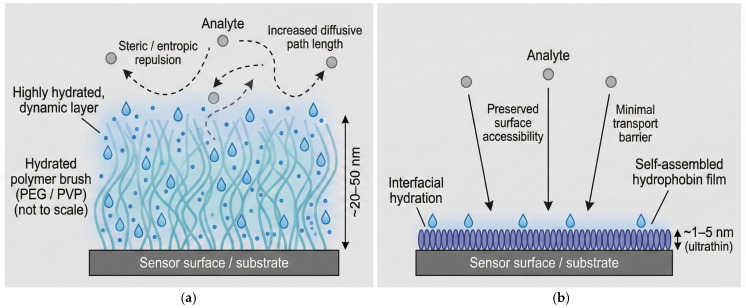
(**a**) Polymer brush coatings form thick, hydrated layers of densely grafted polymer chains that resist fouling through steric and entropic repulsion but may impede near-surface transport. (**b**) Hydrophobin coatings self-assemble into ultrathin amphiphilic protein films that modify interfacial properties while preserving surface accessibility and minimizing diffusion barriers.

These limitations have prompted increasing interest in antifouling strategies that decouple fouling resistance from thick, highly hydrated polymer layers. In particular, ultrathin, self-assembling bio-derived coatings—such as hydrophobin films—offer a fundamentally different architectural paradigm in which interfacial properties are governed by nanometer-scale protein assemblies rather than extended polymer brushes [4,13]. Figure 1 schematically contrasts the structural organization and transport implications of conventional polymer brush coatings with those of hydrophobin-based ultrathin films, highlighting the differences in thickness, hydration profile, and near-surface accessibility that motivate the hydrophobin-focused strategies discussed in the following section [1,19].

## 4. Hydrophobins: Structure, Classification, and Self-Assembly

Hydrophobin-based coatings differ fundamentally from polymer brush systems in that they rely on the spontaneous self-assembly of nanometer-scale amphiphilic protein films rather than extended polymeric barriers. Hydrophobins are a unique class of small, cysteine-rich proteins secreted by filamentous fungi, typically consisting of approximately 100 amino acids and characterized by a conserved pattern of eight cysteine residues forming four disulfide bonds that stabilize their tertiary structure [2,13]. These proteins are abundantly expressed in fungal cell walls, aerial hyphae, and fruiting bodies, where they mediate interactions between fungal tissues and surrounding air–water and solid–liquid interfaces [42,43,44]. Their defining feature is amphiphilicity: distinct hydrophobic and hydrophilic domains enable hydrophobins to spontaneously adsorb and self-assemble at interfaces, dramatically altering surface wettability, adhesion, and surface energy without covalent surface modification or complex grafting strategies [1,4,13,16].

Based on differences in sequence homology, solubility, and supramolecular organization, hydrophobins are broadly classified into two groups: Class I and Class II [13]. Class I hydrophobins undergo a pronounced conformational transition upon self-assembly, forming highly ordered, amyloid-like rodlet structures that are resistant to detergents, extreme pH, and elevated temperatures [14,17]. These assemblies exhibit exceptional mechanical and chemical stability, properties that are biologically advantageous for protecting fungal fruiting bodies from wetting and environmental stress and technologically attractive for long-term or permanently implanted biomedical and sensing applications [34,44]. In contrast, Class II hydrophobins form more soluble, non-amyloid monolayers that retain amphiphilic ordering but can be removed or reorganized using mild solvents or detergents, offering advantages for temporary coatings, disposable sensor platforms, or surfaces that require periodic regeneration [4,13].

The self-assembly of hydrophobins is driven primarily by interfacial energy minimization rather than bulk aggregation. Upon exposure to aqueous environments, hydrophobin molecules orient such that hydrophilic residues face the liquid phase while hydrophobic residues interact with nonpolar surfaces or air, resulting in the spontaneous formation of nanometer-thin amphiphilic films [14,45]. This organization requires no external energy input and produces coatings with thicknesses typically on the order of a few nanometers—substantially thinner than conventional polymer brush or hydrogel-based antifouling systems [13,34]. Such minimal thickness is particularly advantageous for sensor-adjacent surfaces, where excessive coating layers can impede analyte transport, alter local dielectric environments, or attenuate electrochemical and optical signal transduction [3,29].

Importantly, hydrophobins derived from mycelia or fruiting-body sources may exhibit compositional and functional variability depending on species, developmental stage, and extraction context [21,36,37]. While this variability introduces challenges related to reproducibility and standardization, it also suggests opportunities to tailor interfacial properties—such as wettability, friction, and protein resistance—through selective sourcing or processing of fungal biomass [22,23]. For chemosensor and biosensor applications, this raises the prospect of biologically sourced coatings whose interfacial behavior is intrinsically optimized for operation in wet, protein-rich environments [26,46].

Despite these advantages, diverging views remain regarding the practical deployment of hydrophobin coatings. While Class I hydrophobins provide superior durability and resistance to chemical and mechanical stress, their strong intermolecular interactions may limit reversibility and complicate surface regeneration or refunctionalization [17,47]. Conversely, the more dynamic nature of Class II hydrophobins raises questions about long-term stability under continuous fluid flow, repeated sterilization cycles, or extended field deployment [4,48]. Understanding and balancing these trade-offs—particularly in the context of mushroom-derived hydrophobin sources—is therefore essential for selecting appropriate hydrophobin classes and deposition strategies for specific medical device and chemosensor applications.

In the context of this review, the term “mushroom-derived hydrophobins” refers specifically to hydrophobin proteins obtained from fungal fruiting bodies or associated mycelial biomass of macrofungi commonly used in biomaterials and surface science studies. These species differ in both hydrophobin expression profiles and dominant hydrophobin class, which has direct implications for film robustness, reversibility, and tribological behavior. A non-exhaustive overview of representative mushroom species and reported hydrophobin characteristics is provided in Table 1.

## 5. Literature Synthesis: Wettability and Tribological Trends

The antifouling performance of hydrophobin-based coatings arises from their ability to modify surface energy and interfacial wettability through the formation of nanometer-thin amphiphilic films. Across diverse substrates, hydrophobin-treated interfaces have been reported to reduce nonspecific protein adsorption, diminish bacterial adhesion, and decrease platelet attachment relative to unmodified polymer surfaces [1,2,4]. These effects are commonly attributed to interfacial free-energy shifts, altered hydration structure, and the formation of relatively uniform molecular layers that lack strong anchoring sites for biomolecular adsorption, in contrast to the steric repulsion mechanisms of dense polymer brushes [11,13,14]. Because early-stage fouling governs downstream biofilm formation and thrombogenic responses, even partial suppression of conditioning-film formation can yield disproportionate functional benefits for medical devices and sensor-adjacent components [27,28].

Hydrophobin films have also been shown to influence tribological behavior at soft–solid interfaces relevant to biomedical operation. Unlike PEG and PVP coatings, which rely on hydrated polymer-rich boundary layers, hydrophobin coatings can reduce friction through molecularly thin interfacial mechanisms compatible with surface-sensitive devices [4,34]. Under aqueous conditions, amphiphilic hydrophobin films support boundary lubrication by stabilizing interfacial hydration layers and reducing adhesive interactions between contacting surfaces [34,35]. For devices and sensor housings subject to insertion, sliding contact, or repeated handling, friction reduction contributes not only to usability but also to durability by mitigating wear and slowing coating damage under repetitive motion [10,20].

Tribological performance of hydrophobin coatings is strongly condition-dependent. Reported friction outcomes vary with counterface material, applied load, sliding speed, hydration state, and wetting history [17,34]. Assembly morphology further modulates performance: rodlet-rich amyloid structures and more homogeneous monolayers can introduce micro- and nanoscale roughness that either enhances boundary lubrication or exacerbates friction through asperity-driven interactions, depending on film uniformity and mechanical integrity [17,18]. Consequently, tribological behavior often correlates more strongly with deposition protocol and post-treatment history than with hydrophobin identity alone [16,38].

Differences in antifouling and tribological behavior are also associated with hydrophobin class and biological source. From an application-design perspective, Class I hydrophobins, which assemble into mechanically robust amyloid-like rodlet structures, generally exhibit greater chemical and mechanical stability and retain function under shear or prolonged exposure [14,18]. In contrast, Class II hydrophobins form more dynamic and reversible films that may facilitate regeneration or disposability but are more susceptible to displacement under continuous flow or harsh sterilization conditions [1,48]. These class-dependent differences therefore represent a practical materials-selection trade-off between long-term durability and surface regenerability in sensor and biomedical interface applications. For mushroom-derived hydrophobins, additional complexity arises from multiple isoforms and residual co-extractives in native extracts, which can alter assembly kinetics, wettability shifts, and friction behavior relative to recombinant single-protein systems [19,22,23]. Accordingly, careful reporting of extraction protocols, purification level, coating mass or surface coverage, and baseline surface characterization is essential when interpreting tribological outcomes from biomass-derived coatings [38,61].

Overall, the literature indicates that hydrophobin films provide antifouling and lubricating behavior through mechanisms distinct from conventional synthetic polymer coatings, with the practical advantages of nanometer-scale thickness and spontaneous self-assembly [4,13]. For sensor-adjacent deployment, the principal challenges concern maintaining durable and reproducible performance under operational stresses such as flow, wear, sterilization, and storage, and achieving sufficient standardization of mushroom-derived hydrophobin systems for comparative evaluation and translation [20,38]. To contextualize these literature-reported trends in a controlled sensor-adjacent configuration, representative pilot observations are presented in the following section.

## 6. Representative Observations (Pilot Illustration)

This section presents representative experimental observations drawn from mushroom-derived hydrophobin coatings to contextualize the antifouling and tribological mechanisms discussed in the preceding sections. These observations are included to provide a practical illustration of hydrophobin-mediated surface modification and to demonstrate qualitative consistency with trends reported in the existing literature. They are not intended to establish new performance benchmarks, rank hydrophobin sources, or claim novel mechanisms, but rather to ground the review discussion in experimentally observed behavior relevant to sensor-adjacent surfaces. Detailed experimental procedures, measurement conditions, and additional documentation of the tribological testing system are provided in the Supporting Information S1–S5.

### 6.1. Mushroom-Derived Hydrophobin Sources and Extraction Context

**Figure 2:**
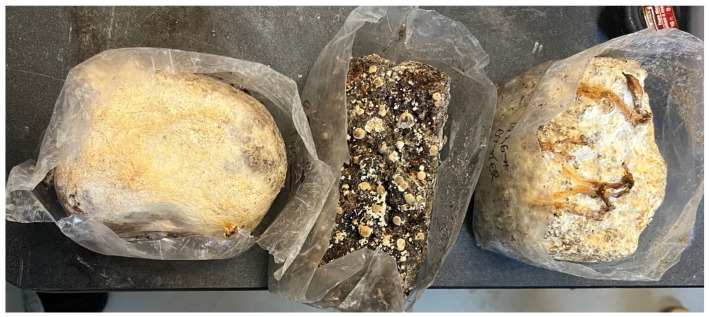
From left to right: Lion’s Mane, Turkey Tail, and Oyster mushroom bricks used for the extraction process.

Hydrophobin-containing extracts were obtained from Lion’s Mane (*Hericium erinaceus*), Turkey Tail (*Trametes versicolor*), and Oyster mushroom (*Pleurotus ostreatus*), three mushroom species representative of distinct fungal taxa commonly investigated in biomaterial and surface-functionalization studies. Raw Material is illustrated in Figure 2.

The selected mushroom species—*Pleurotus ostreatus* (Oyster mushroom), *Trametes versicolor* (Turkey Tail), and *Hericium erinaceus* (Lion’s Mane)—were chosen because they are widely cultivated macrofungi with documented hydrophobin expression and established relevance in biomaterials and surface-functionalization research. In particular, hydrophobin systems from *Pleurotus ostreatus* have been extensively investigated in prior surface-science studies, providing a useful benchmark for comparison, while the inclusion of taxonomically distinct species enables qualitative comparison across different mushroom-derived hydrophobin sources.

**Figure 3:**
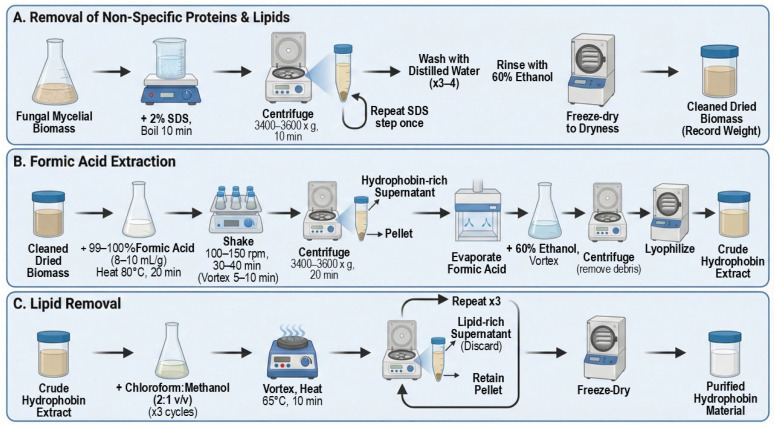
Schematic illustration of hydrophobin extraction and preparation workflow used to obtain surface-active protein fractions.

A schematic overview of the mushroom sources and the corresponding extraction workflow is shown in Figure 3, highlighting the use of aqueous and solvent-assisted steps to recover surface-active protein fractions. The resulting hydrophobin-containing solutions exhibited visible interfacial activity, consistent with the amphiphilic nature of hydrophobins reported in prior studies [2,47].

Although extraction protocols and purity levels can influence film formation and surface behavior, the present observations are intended to reflect typical outcomes achievable from mushroom-derived hydrophobin preparations, rather than optimized or standardized formulations. Variations between species are therefore interpreted in the context of known differences in hydrophobin composition, isoform distribution, and self-assembly propensity [25,49,54].

### 6.2. Surface Wettability Modification

Hydrophobin-containing coatings derived from the three mushroom sources were applied to polymeric substrates commonly used in medical devices and sensor housings. Following coating application, qualitative and quantitative changes in surface wettability were observed. Hydrophobin self-assembly at the solid–liquid interface leads to surface energy modification through the orientation of hydrophilic residues toward the aqueous phase and hydrophobic domains toward the substrate, a mechanism widely described for fungal hydrophobins [13,38,62].

**Figure 4:**
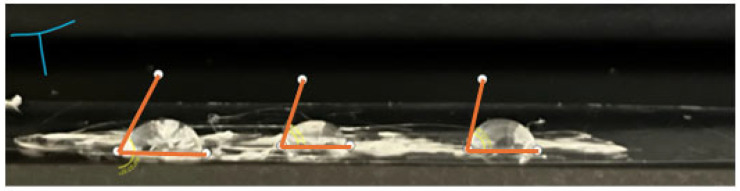
Representative contact-angle measurements indicated increased hydrophobicity of PET substrates following coating with mushroom-derived hydrophobin preparations compared with uncoated PET (60.33° ± 4.62). From left to right: Lion’s mane, Oyster Mushroom, and Turkey Tail coating. Coated surfaces exhibited contact angles of approximately 73–74° for Lion’s Mane and Oyster-derived coatings and ~71° for Turkey Tail coatings (detailed measurements are provided in the Supporting Information, Table S1).

Representative water contact angle measurements obtained after hydrophobin treatment are shown in Figure 4. In all cases, coated surfaces exhibited a measurable shift toward increased wettability relative to unmodified substrates. While absolute contact angle values varied among coatings derived from different mushroom species, the overall trend is consistent with previously reported effects of both Class I and Class II hydrophobins on polymeric surfaces [63]. These differences are attributed to variations in hydrophobin composition and film organization (described in Table 2) rather than to fundamentally distinct wetting mechanisms.

Contact-angle measurements were obtained using a profilometer-based droplet profile analysis method, averaging 5 droplets, which reduces optical distortion effects associated with off-axis imaging. Reported values represent representative measurements intended to illustrate wettability trends rather than formal statistical characterization (See Supplemental Material Section S4 for additional details).

### 6.3. Tribological Behavior Under Hydrated Conditions

**Figure 5:**
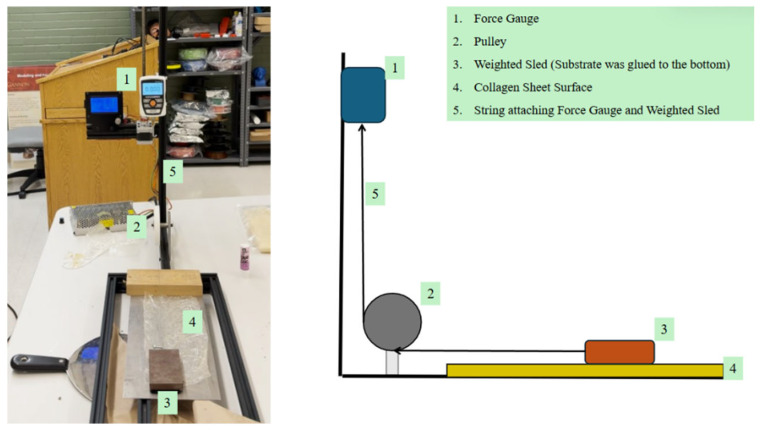
The figure above shows the friction tester that was used for this experiment and a simplified model of the tester.

To further illustrate the functional consequences of hydrophobin-mediated surface modification, representative friction measurements were conducted under hydrated conditions. Tribological testing was conducted using a custom-built linear sliding tribometer based on a modified Prusa 3D printer platform, equipped with an in-line force sensor for direct measurement of friction force. A schematic and photograph of the testing apparatus are shown in Figure 5.

The testing configuration consisted of a stainless steel base plate rigidly mounted on the printer bed and a steel slider block attached to the moving carriage. Collagen film layers were applied to the base plate while the slider block was coated with hydrophobin from the different mushroom species. The tribo-pair was then a collagen–hydrophobin contact interface, wetted with deionized water, representative of soft biological sliding conditions.

The slider block was translated horizontally across the collagen-coated base plate under a constant normal load determined solely by the weight of the slider assembly. The applied normal load was 4.448 N (1 lbf) and remained constant for all tests. No external loading or active normal force control was employed, ensuring a gravity-defined, repeatable contact condition.

Friction force was measured inline along the direction of motion, such that the recorded force corresponded directly to resistance arising from interfacial sliding.

**Figure 6:**
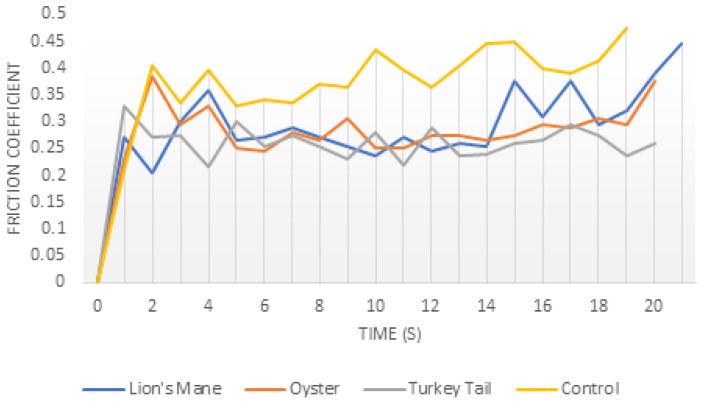
Representative coefficient of frictions as a function of time for hydrophobin coatings derived from three mushroom species under hydrated conditions. (Illustrative comparison; not intended as performance ranking).

As summarized in Figure 6, hydrophobin-coated surfaces exhibited reduced interfacial friction when compared to uncoated collagen-collagen substrates across coatings derived from the three mushroom species examined. The time-dependent friction response shown in Figure 6 highlights a characteristic reduction in frictional resistance following hydrophobin coating, consistent with boundary lubrication facilitated by thin, hydrated interfacial films. Similar friction-reducing effects have been reported for hydrophobin-coated polymer and solid surfaces in aqueous environments and are generally attributed to reduced adhesive interactions and stabilized hydration layers rather than bulk lubrication effects [56,66,67].

Differences in frictional response among mushroom-derived coatings were observed; however, these variations align with previously reported class- and species-dependent differences in hydrophobin film robustness, morphology, and self-assembly behavior [56,66,67]. Importantly, these measurements are not presented as definitive quantitative comparisons of tribological performance. Frictional outcomes depend strongly on substrate material, coating uniformity, hydration state, applied load, sliding velocity, and measurement protocol, limiting direct cross-system comparison.

### 6.4. Contextual Interpretation and Relevance to Sensor-Adjacent Surfaces

Taken together, these representative observations support the mechanistic interpretations discussed in Section 4 and Section 5 by providing experimental context for hydrophobin-mediated wettability control and friction reduction. The observed trends in contact angle modification and hydrated tribological behavior are consistent with the broader literature on fungal hydrophobins and reinforce their potential relevance for medical device and sensor-adjacent surface engineering.

From a chemosensor perspective, the combination of nanometer-scale film thickness, spontaneous self-assembly, and measurable reductions in friction and surface energy highlights the appeal of mushroom-derived hydrophobin coatings as interface-modifying layers that can influence fouling and mechanical interactions without imposing thick polymer barriers. Nevertheless, comprehensive performance evaluation, long-term stability assessment, and application-specific optimization remain essential topics for future investigation.

## 7. Implications for Chemosensors and Biosensors

The surface properties of chemosensors and biosensors play a critical role in determining analytical performance, long-term stability, and reliability in complex biological environments. Nonspecific adsorption of proteins, cells, or microorganisms at the sensing interface can induce signal drift, reduced sensitivity, increased noise, and compromised selectivity, particularly for sensors operating in blood, serum, or other biofluids [3,8]. Because fouling-related degradation often evolves gradually rather than catastrophically, it can be difficult to distinguish from genuine analyte-driven signal changes, underscoring the importance of antifouling surface engineering as a central design consideration in sensor development.

Hydrophobin-based coatings exhibit several characteristics that are particularly relevant for chemosensor and biosensor interfaces. Their ability to form nanometer-scale, self-assembled films enables modulation of surface wettability and interfacial energy without introducing the thick, highly hydrated polymer layers typical of PEG- or PVP-based coatings. This minimal thickness is advantageous for surface-sensitive detection modalities—including electrochemical, optical, and mass-based sensors—where analyte transport, electric field distribution, or near-surface refractive index changes directly govern signal generation [15,46]. By reducing nonspecific adsorption while preserving near-surface accessibility, hydrophobin films may help maintain sensor responsiveness and calibration stability over extended operating periods.

Coating thickness and internal structure also influence mass transport and signal kinetics in surface-sensitive chemosensors. In polymer-brush systems such as PEG or PVP, hydrated layers often extend tens of nanometers from the surface and exhibit dynamic chain motion, introducing an additional diffusive resistance for small molecules and ions approaching the sensing interface [12,35]. While modest in bulk systems, this resistance can become significant for electrochemical, plasmonic, or impedance-based sensors where signal generation depends on rapid near-surface transport.

In contrast, self-assembled hydrophobin monolayers are typically only a few nanometers thick and lack extended, mobile polymer chains. As a result, analyte diffusion toward the sensing interface occurs over a substantially shorter path length, potentially preserving faster response times and reducing signal lag [15,68]. Although systematic quantitative comparisons remain limited, this structural distinction provides a mechanistic rationale for the observed compatibility of hydrophobin coatings with surface-sensitive sensor modalities.

Representative wettability trends discussed in Section 6 further suggest that mushroom-derived hydrophobin coatings can modulate surface energy in a controlled manner across polymeric substrates commonly used in sensor housings and microfluidic components. Such tunability is relevant not only for antifouling, but also for managing sample wetting, bubble formation, and fluid spreading—factors that strongly influence reproducibility in microfluidic and point-of-care sensing platforms [12,69]. Importantly, these effects are achieved without covalent modification of the sensing surface, supporting compatibility with existing sensor architectures and fabrication workflows [16].

In addition to antifouling performance, the tribological behavior of hydrophobin coatings has implications for sensor durability and reliability. Reduced interfacial friction under hydrated conditions may mitigate mechanical degradation of sensor surfaces, protective membranes, and microfluidic channels exposed to fluid flow or repeated handling [32,34]. For wearable, implantable, or portable point-of-care sensors, mechanical stresses and biofouling often act synergistically, with fouling-induced friction accelerating wear. By moderating both fouling and friction, hydrophobin coatings may help interrupt this feedback loop and extend functional device lifetime.

Hydrophobin class and biological source further influence suitability for sensor integration. Class II hydrophobins, which form more dynamic and reversible films, may be advantageous for disposable, regenerable, or periodically recalibrated sensor platforms where surface renewal is desirable [1,4]. Conversely, the greater mechanical and chemical robustness of Class I hydrophobin films may better support long-term or continuously operating sensors, provided that film irreversibility does not impede analyte access or regeneration strategies [23,44]. In mushroom-derived systems, species-dependent composition and assembly behavior introduce additional design flexibility but also necessitate careful characterization to ensure reproducibility across sensor batches [21,70].

Despite these advantages, challenges remain before hydrophobin-based coatings—particularly those derived from mushroom biomass—can be broadly adopted in chemosensor technologies. Long-term stability under continuous flow, compatibility with fabrication and sterilization processes, and consistency across substrates and extraction batches require further investigation [48,71]. Moreover, systematic studies directly linking hydrophobin film morphology, thickness, and composition to sensor performance metrics such as sensitivity, drift, and response time are still limited. Nevertheless, the combination of antifouling efficacy, minimal coating thickness, and favorable tribological and wetting properties positions hydrophobins as promising candidates for next-generation chemosensor and biosensor surface engineering [13,26]. A feature comparison between hydrophobin and PEG/PVP coatings is provided in Table 3.

## 8. Discussion

### 8.1. Antifouling as a Mechanism for Signal Stability

The preceding sections indicate that, for chemosensors and biosensors, antifouling performance is most usefully interpreted in terms of signal stability over time rather than simple suppression of biological adhesion. In surface-sensitive platforms, early-stage nonspecific adsorption promotes conditioning-film formation that alters interfacial chemistry and transport pathways, leading to baseline drift, increased noise, and gradual loss of calibration fidelity [3,30]. By modulating surface energy and hydration behavior at the nanometer scale, hydrophobin coatings act at the onset of this fouling cascade, stabilizing sensor response through direct control of the physicochemical interface rather than downstream signal processing [4,13].

### 8.2. Surface Accessibility and Ultrathin Interfacial Design

Surface accessibility constitutes a second design axis along which hydrophobin-based coatings differ fundamentally from conventional polymeric antifouling strategies. PEG- and PVP-based systems rely on hydrated polymer brush layers that can extend tens of nanometers from the surface and exhibit significant chain mobility, introducing additional diffusive resistance and potentially perturbing near-surface electric fields or optical interactions [8,12]. In contrast, hydrophobins self-assemble into ultrathin amphiphilic films—typically only a few nanometers thick—that modify interfacial properties without introducing extended polymeric barriers [13,16]. For surface-sensitive transduction modalities, this minimal thickness preserves analyte access, maintains rapid response kinetics, and reduces attenuation of electrochemical, optical, or impedance-based signals governed by near-surface interactions [15,68]. Hydrophobin coatings can therefore be viewed as interface-preserving modifications rather than conventional antifouling layers.

### 8.3. Friction, Wear, and the Mechanical Basis of Durability

In many sensor-adjacent environments, durability is governed less by chemical stability than by friction-driven wear under hydrated conditions. Sensors exposed to fluid flow, microfluidic confinement, handling, or mechanical interaction experience repeated interfacial shear stresses that can promote surface damage, coating thinning, and accelerated fouling over time [10,34]. Within this framework, the friction-reducing behavior reported for hydrophobin-coated surfaces provides a direct mechanistic link between tribological performance and functional durability. By lowering adhesive and shear forces, reduced friction mitigates wear-driven degradation and helps preserve surface integrity, offering a physically grounded pathway for extending sensor lifetime without reliance on thick sacrificial layers [4,34].

### 8.4. Electrode Interfaces as a Case Study for Chemosensors

Electrodes provide a particularly instructive case for examining the combined implications of hydrophobin coatings on signal stability, surface accessibility, and durability. In electrochemical chemosensors and biosensors, the electrode–electrolyte interface governs signal transduction through near-surface electron-transfer kinetics, interfacial chemistry, and mass transport. Consequently, even nanometer-scale modifications can produce measurable effects on sensitivity, baseline stability, and response time [15,68]. Hydrophobins have been shown to self-assemble directly on conductive substrates, including gold electrodes, forming stable ultrathin films that preserve electrochemical functionality while modifying interfacial properties [15]. This distinguishes hydrophobin coatings from thicker polymeric antifouling layers, which can partially screen electrode surfaces, increase charge-transfer resistance, or introduce additional diffusion barriers [12].

From a signal-stability perspective, electrode fouling is a dominant contributor to baseline drift and sensitivity loss in electrochemical sensors operating in complex media [3]. By reducing nonspecific adsorption at the electrode surface, hydrophobin coatings can mitigate conditioning-film formation that progressively alters electron-transfer pathways. Under flowing or mechanically dynamic conditions, friction reduction at the solid–liquid interface further provides a plausible mechanism for preserving electrode integrity and electrochemical performance during extended operation [4,34].

### 8.5. Design Trade-Offs and Translational Considerations

Differences between hydrophobin classes further refine the durability–function trade space. Class I hydrophobins form mechanically robust, chemically resistant assemblies well suited for prolonged shear, continuous flow, or repeated hydration cycles in long-term sensing applications [14,44]. In contrast, the greater reversibility of Class II hydrophobin films may be advantageous for disposable, regenerable, or periodically recalibrated sensor platforms, where surface renewal is desirable [17,48]. This class-dependent behavior thus represents a tunable design parameter that can be matched to sensor lifecycle and deployment conditions.

Mushroom-derived hydrophobins occupy a distinctive position within this design framework. Their biological origin provides access to naturally evolved surface-active proteins compatible with aqueous, protein-rich sensing environments [19,20]. At the same time, species-dependent composition and extraction context introduce variability that must be addressed through careful characterization to ensure reproducibility and integration with sensor fabrication workflows [21,38]. These considerations underscore the need for standardized testing and application-specific optimization prior to widespread translational adoption.

### 8.6. Biological and Regulatory Considerations Further Motivate Continued Investigation

Although hydrophobins are naturally occurring fungal proteins, their long-term biocompatibility, immunogenic potential, and behavior under chronic exposure conditions require thorough evaluation, particularly for implantable or continuously operating biosensors [2,32,72]. These issues are especially pertinent when transitioning from well-defined recombinant hydrophobins to biomass-derived preparations, where compositional complexity may influence biological response. For implantable applications, translational deployment would therefore require application-appropriate biocompatibility testing (e.g., cytotoxicity, sensitization, irritation, implantation, and hemocompatibility studies) together with controlled purification and manufacturing processes to ensure batch consistency and sterility. In this context, recombinant production or highly purified hydrophobin preparations may provide practical advantages for regulatory qualification compared with crude biomass-derived extracts.

## 9. Limitations and Future Research Directions

Despite the promising antifouling, wetting, and interfacial properties discussed in this review, several limitations must be acknowledged for hydrophobin-based surface modifications, particularly in chemosensor and biosensor applications. Reported antifouling and tribological performance varies substantially with hydrophobin class, fungal source, substrate material, and deposition conditions. Differences in protein composition, assembly morphology, surface coverage, and post-deposition treatment can produce divergent outcomes even among nominally similar systems, complicating direct comparison across studies. This variability highlights the need for standardized testing protocols, reporting metrics, and surface characterization practices to enable meaningful cross-study evaluation and reproducibility [1,4,13,38].

Although mushroom-derived hydrophobins provide an accessible and potentially sustainable source of surface-active proteins, scalability considerations remain important for industrial deployment. Extraction from fungal biomass can enable low-cost production using agricultural substrates; however, variability in protein yield, isoform distribution, and purification efficiency may limit large-scale consistency. Recombinant production systems, including bacterial and yeast expression platforms, offer an alternative pathway capable of producing defined hydrophobin isoforms at an industrial scale with improved batch reproducibility, albeit often at a higher upstream processing cost. Future industrial applications may therefore adopt hybrid strategies in which naturally derived hydrophobins support exploratory materials development, while recombinant production enables standardized manufacturing for high-performance sensing and biomedical devices.

Long-term stability represents a second critical limitation, particularly for sensor platforms operating under continuous flow, cyclic mechanical loading, or repeated exposure to cleaning and sterilization processes. While Class I hydrophobins exhibit high chemical and mechanical robustness due to their amyloid-like rodlet structures, strong intermolecular interactions may limit surface regeneration, refunctionalization, or adaptive tuning after deployment [23,34,44]. Conversely, the greater reversibility of Class II hydrophobin films raises concerns regarding durability under extended operational conditions, including shear-induced desorption, gradual rearrangement, or displacement by surface-active components in biological fluids [13,17,48]. Systematic studies that quantitatively compare stability–reversibility trade-offs across application-relevant time scales and operating environments remain limited, particularly for sensor-adjacent interfaces.

From a translational and manufacturing perspective, integration of hydrophobin coatings into sensor fabrication workflows presents additional challenges. Compatibility with microfabrication steps such as photolithography, metallization, bonding, and device packaging must be demonstrated across diverse substrate chemistries prior to widespread adoption [15,16,46]. For mushroom-derived hydrophobin systems, these challenges are compounded by batch-to-batch variability, co-extracted impurities, and species-dependent composition, all of which can influence assembly behavior and interfacial performance unless extraction and purification protocols are carefully controlled and reported [19,21,70]. Scalability of protein production and purification—whether via fungal cultivation, biomass extraction, or recombinant expression—also remains a practical consideration for commercial sensor deployment [71,73].

Biological and regulatory considerations further motivate continued investigation. Although hydrophobins are naturally occurring fungal proteins, their long-term biocompatibility, immunogenic potential, and behavior under chronic exposure conditions require thorough evaluation, particularly for implantable or continuously operating biosensors [2,32,72]. These issues are especially pertinent when transitioning from well-defined recombinant hydrophobins to biomass-derived preparations, where compositional complexity may influence biological response.

Future research should therefore prioritize several complementary directions. These include the development of standardized antifouling and tribological characterization methodologies tailored to sensor-relevant conditions; systematic elucidation of structure–function relationships across hydrophobin classes, fungal species, and assembly states; and long-term performance assessments under realistic flow, wear, and biofouling environments [4,20,26]. Advances in protein engineering, controlled fungal cultivation, and hybrid coating strategies may further enable the development of hydrophobin-based interfaces optimized for specific sensing modalities [38,74,75].

Collectively, these efforts will be essential to transition hydrophobin-based—and particularly mushroom-derived—coatings from promising laboratory demonstrations to robust, reproducible, and scalable solutions for next-generation chemosensor and biosensor technologies.

## 10. Conclusions

This review has examined hydrophobin-based antifouling and anti-wetting surface modifications with specific emphasis on their relevance to medical devices and chemosensor-adjacent applications. By synthesizing the current literature and contextualizing representative experimental observations, the work highlights how hydrophobins offer a fundamentally different, bio-derived alternative to conventional synthetic polymer coatings such as PEG and PVP. Rather than relying on thick, highly hydrated polymer brushes, hydrophobins spontaneously self-assemble into nanometer-scale amphiphilic films that modulate surface wettability, biological adhesion, and interfacial friction while preserving near-surface accessibility—an essential requirement for surface-sensitive sensing modalities.

From a chemosensor perspective, this combination of properties is particularly compelling. Minimal coating thickness reduces the risk of attenuating electrochemical, optical, or mass-based signals, while the ability to tune interfacial energy and hydration behavior supports mitigation of nonspecific adsorption and signal drift in complex biofluids. The representative wettability and tribological trends discussed in this review illustrate how hydrophobin-mediated surface modification can simultaneously influence fouling resistance and mechanical interaction under hydrated conditions, addressing two failure modes that often act synergistically to limit sensor lifetime and reliability.

Importantly, this review also underscores that hydrophobin performance is not monolithic. Differences between Class I and Class II hydrophobins, and variability associated with fungal species and sourcing, introduce meaningful trade-offs between robustness, reversibility, and long-term stability. In this context, mushroom-derived hydrophobins emerge as a particularly interesting avenue: they offer access to naturally evolved surface-active proteins from renewable and potentially scalable biomass, while also introducing challenges related to compositional variability and standardization. Rather than viewing this variability as a limitation alone, it may also represent an opportunity to tailor interfacial properties through controlled selection of fungal sources, processing conditions, or hybrid coating strategies.

At the same time, significant challenges remain before hydrophobin-based coatings—whether recombinant or mushroom-derived—can be broadly adopted in practical chemosensor technologies. These include the need for standardized characterization methodologies, systematic assessment of long-term stability under realistic operating conditions, compatibility with sensor fabrication and sterilization workflows, and improved understanding of biological responses during prolonged exposure. Addressing these challenges will require coordinated efforts spanning surface science, protein engineering, sensor design, and translational manufacturing.

Taken together, the collective evidence reviewed here supports the view that hydrophobins constitute a versatile and promising platform for antifouling surface engineering in chemosensors and biosensors. Their unique combination of self-assembly, ultrathin film formation, and multifunctional interfacial behavior positions them as a complementary—and in some cases enabling—alternative to conventional polymer-based coatings. Continued research focused on reproducibility, durability, and application-specific integration will be essential to transition hydrophobin-based interfaces from laboratory demonstrations to robust components of next-generation sensing technologies.

## Figures and Tables

**Table 1:** Reported hydrophobin characteristics for representative mushroom species.

Organisms	Class	Extraction Method	Yield Efficiency	Application Potential
*Schizophyllum commune* [34,42]	Class I (SC3)	Organic solvent extraction, hydrophobic chromatography	Moderate	Catheter coatings, biosensors
*Pleurotus ostreatus* [21,22,25,36,49,50,51,52,53,54,55]	Class I	Ethanol washing, centrifugation	High	Medical device coatings, antifouling films
*Trichoderma reesei* [56,57,58,59,60]	Class II (HFBI)	Foam Fractionation (CO_2_)	High	Industrial coatings, emulsifiers

**Table 2:** Hydrophobin Composition for the different mushroom analyzed as extracted from the literature.

Feature	Oyster Mushroom (*P. ostreatus*)	Turkey Tail (*T. versicolor*)	Lion’s Mane (*H. erinaceus*)
Primary Hydrophobins Genes	VMH2, VMH3, POH1, POH2, POH3 [48,51]	Over 40 Genes [64]	Surface-active protein HEP3 [65]
Protein Class	Class I and II	Class I (highly robust films)	Likely Class I (structural)
Surface Activity	High; shifts contact angles by >30°	Very high; contact angles achievable 105–120°	Moderate; focus is often on bioactivity
Stability	Resistant to SDS and heat	Resistant to oxygen, oil, and water	Evaluated for immunomodulation
Primary Context	Standard for surface science research	Bio-packaging and water barriers	Medicinal and bioactive coatings

**Table 3:** Feature comparison between Hydrophobin and PEG/PVP Coating.

Property	PEG/PVP Coatings	Hydrophobin Films
Typical thickness	~20–100 nm	~2–10 nm
Wetting shift (Δθ)	20–60° (substrate dependent)	20–50°
Friction reduction (hydrated)	Moderate to high	Moderate
Assembly mechanism	Grafted/adsorbed polymer	Self-assembled protein
Reversibility	Limited	Class-dependent

## Data Availability

The original contributions presented in this study are included in the article/Supplementary Material. Further inquiries can be directed to the corresponding author.

## Supplementary Materials

The following supporting information can be downloaded at https://www.mdpi.com/article/10.3390/s26051642/s1. Section S1: Mushroom Sources and Hydrophobin Extraction Context; Section S2: Hydrophobin Extraction Procedure; Section S3: Hydrophobin Coating Deposition Procedure; Section S4: Contact Angle Measurement Procedure; Section S5: Tribological Measurement System and Calibration.
