# Supplementary material for: Mushroom-Derived Hydrophobins for Antifouling and Interface Preservation in Chemosensors
Source: Sensors (Basel). 2026 Mar 5;26(5):1642. doi: 10.3390/s26051642 (PMC12986670; doi:10.3390/s26051642)
Supplement: Supplementary file 1 [file sensors-26-01642-s001.zip › sensors-4103940-supplementary.pdf]

Review

Mushroom-Derived Hydrophobins for Antifouling and Interface Preservation in Chemosensors

Nardos F. Bisrat [1], Bethany R. Finnefrock [1], Matthew D. Gacura [2], Longyan Chen [1] and Davide Piovesan [1,*]

[1] Biomedical, Industrial and Systems Engineering Department, Gannon University, 109 University Square, Erie, PA 16541, USA; bisrat001@gannon.edu (N.F.B.); finnefro002@gannon.edu (B.R.F.); chen084@gannon.edu (L.C.)
[2] Biology Department, Gannon University, 109 University Square, Erie, PA 16541, USA; gacura001@gannon.edu
* Correspondence: piovesan001@gannon.edu; Tel.: +1-814-871-7221

S1. Mushroom Sources and Hydrophobin Extraction Context

Hydrophobin-containing protein preparations were derived from three widely cultivated macrofungal species: Pleurotus ostreatus (Oyster mushroom), Trametes versicolor (Turkey Tail), and Hericium erinaceus (Lion's Mane). These species were selected because they represent taxonomically distinct fungal groups commonly investigated in biomaterials and surface-functionalization studies and are reported to express multiple hydrophobin isoforms in genomic and proteomic investigations.

For extraction-based preparations, fungal biomass was obtained either from laboratory-cultured mycelial growth or commercially available fruiting-body material processed into compressed biomass blocks. Biomass was mechanically fragmented into small pieces to increase solvent accessibility and facilitate subsequent extraction steps. Prior to extraction, samples were stored at refrigerated or frozen conditions to minimize protein degradation and preserve surface-active protein fractions.

All biomass handling procedures were conducted using clean laboratory glassware and solvent-compatible containers to prevent contamination that could affect interfacial activity measurements. The prepared biomass served as the starting material for the hydrophobin extraction procedure described in Section S2.

S2. Hydrophobin Extraction Procedure

Hydrophobin-containing protein extracts were prepared using a detergent-stripping, acid-extraction, and lipid-removal workflow adapted from established fungal hydrophobin isolation procedures. The sequence of operations followed the station-by-station process illustrated in Figure 3, reported here as Figure S1 for ease of access.

S2.1. Removal of Non-Specific Proteins and Lipids

Dried fungal mycelial biomass was first subjected to detergent stripping to remove loosely bound proteins, lipids, and extracellular materials. Biomass was suspended in 2% (w/v) sodium dodecyl sulfate (SDS) solution at an approximate ratio of 10 mL solution per gram wet biomass and incubated in a boiling water bath for 10 min. Samples were then centrifuged (3,400–3,600 × g, 10 min), and the supernatant was discarded. The SDS treatment was repeated once using fresh detergent solution. Following detergent removal, biomass was rinsed three to four times with distilled water until residual foaming disappeared and then washed once with 60% (v/v) ethanol to remove remaining detergent traces. The cleaned biomass was subsequently freeze-dried to obtain dried material for acid extraction.

S2.2. Formic Acid Extraction of Hydrophobins

Lyophilized biomass was immersed in concentrated formic acid (99–100%) at an approximate solvent-to-solid ratio of 8–10 mL g^{-1} and incubated at approximately 80 °C for 20 min to solubilize hydrophobin proteins. Samples were then agitated under gentle mixing (100–150 rpm) for approximately 30–40 min with intermittent vortexing to improve extraction efficiency. After extraction, suspensions were centrifuged (3,400–3,600 × g, 20 min), and the hydrophobin-rich supernatant was collected. Formic acid was evaporated under a fume hood to dryness, after which the residue was redissolved in 60% ethanol and centrifuged to remove insoluble debris. The clarified supernatant was lyophilized to yield crude hydrophobin extract.

S2.3. Lipid Removal

To remove residual lipids, the crude extract was subjected to three sequential chloroform–methanol (2:1 v/v) washing cycles. For each cycle, the extract was mixed with the solvent system, vortexed, and incubated at approximately 65 °C for 10 min, followed by centrifugation (3,400–3,600 × g). The lipid-rich supernatant was discarded, and the precipitated material was retained. After the third cycle, the resulting precipitate was freeze-dried to obtain purified hydrophobin-containing material.

S2.4. Final Preparation

Purified hydrophobin-containing material was redissolved in distilled water or 60% ethanol and adjusted to near-neutral pH (approximately pH 7) using dilute ammonia when required. Brief heating at approximately 80 °C was optionally applied to promote rodlet self-assembly prior to coating experiments. The resulting preparations are referred to as hydrophobin-containing extracts, as purification to isolate individual hydrophobin isoforms was not performed.

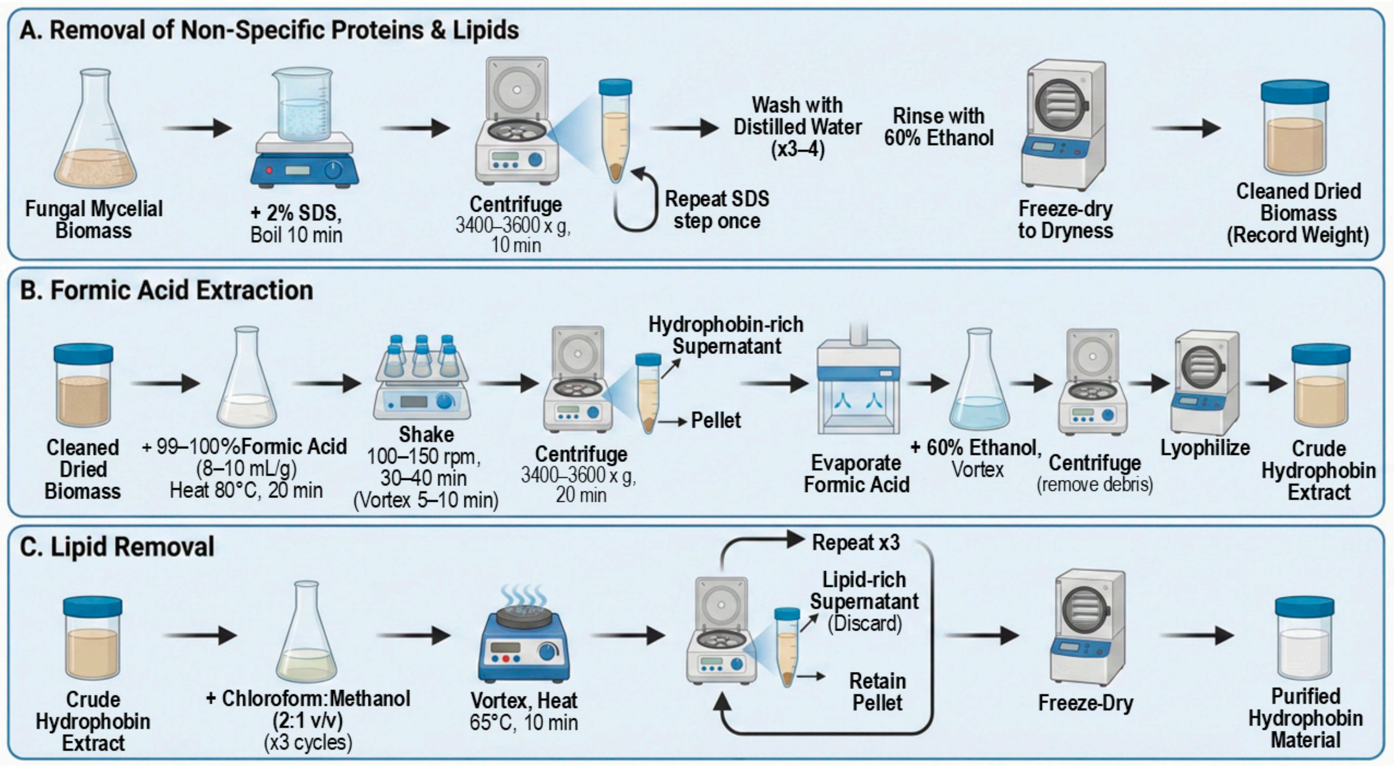

Figure S1. Schematic illustration of hydrophobin extraction and preparation workflow used to obtain surface-active protein fractions.

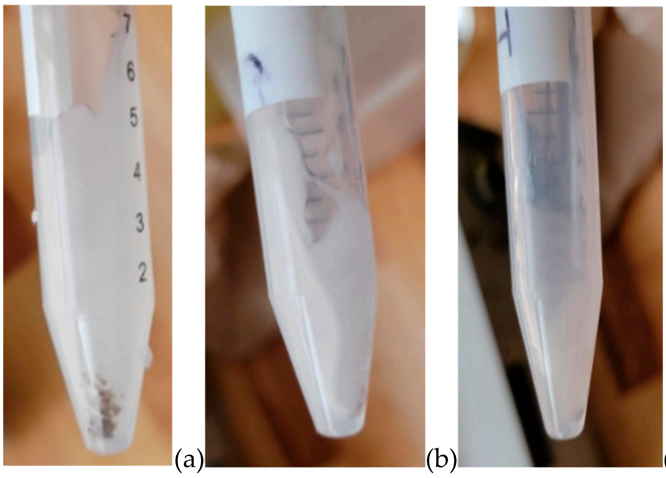

Figure S2. Hydrophobin extracts obtained from mushroom-derived biomass after purification and lyophilization. (a) *Pleurotus ostreatus* (Oyster mushroom) extract; (b) *Hericium erinaceus* (Lion's Mane) extract; (c) *Trametes versicolor* (Turkey Tail) extract.

Hydrophobins were successfully extracted from oyster (*Pleurotus ostreatus*), lion's mane (*Hericium erinaceus*), and turkey tail (*Trametes versicolor*) using SDS stripping, formic acid extraction, and chloroform–methanol lipid removal. After lyophilization, all extracts formed gel-like solid materials, consistent with the aggregation behavior characteristic of Class I hydrophobins. The extracted materials were subsequently rehydrated and applied to PET film substrates to evaluate surface-modification effects. All coatings dried uniformly and adhered well to the polymer surface. Uncoated PET films served as control substrates for wettability and friction measurements.

S3. Hydrophobin Coating Deposition Procedure

Hydrophobin coatings were deposited onto polyethylene terephthalate (PET) film substrates using a direct adsorption and surface-spreading method designed to simulate low-complexity coating processes applicable to polymeric sensor or device materials.

Prior to coating, PET sheets were cleaned with ethanol and rinsed with distilled water to remove surface contaminants and allowed to dry under ambient laboratory conditions. Crude hydrophobin extracts obtained after purification and lyophilization were rehydrated in deionized water to produce a viscous protein suspension suitable for coating.

A measured volume of the hydrophobin suspension was applied directly onto the PET substrate surface. The material was spread uniformly across the film using a sterile spatula or applicator blade to promote surface coverage and facilitate adsorption of hydrophobin molecules at the polymer–air interface. Coated films were then allowed to dry under ambient laboratory conditions (22–25 °C, 30–50 % relative humidity) for a minimum of 12 h to permit solvent evaporation and spontaneous self-assembly of hydrophobin films.

Following drying, coated PET strips were visually inspected to confirm macroscopic film formation and were subsequently cut into standardized dimensions for wettability and tribological characterization. Uncoated PET samples were prepared using the same cleaning procedure and served as experimental controls.

The coating method relies on the intrinsic amphiphilic self-assembly of hydrophobin proteins, which spontaneously organize into interfacial films during drying without the need for cross-linking reagents, surface primers, or thermal curing.

S4. Contact Angle Measurement Procedure

Surface wettability was evaluated using static water contact-angle measurements obtained through droplet profile analysis. Prior to measurement, coated and uncoated polyethylene terephthalate (PET) substrates were cleaned of loose particulates and equilibrated under laboratory ambient conditions (approximately 22–25 °C and 30–50% relative humidity).

A calibrated micro-syringe was used to deposit five independent droplets per sample of deionized water (typically 3–5 μL) onto the substrate surface. Droplets were placed at multiple spatially separated locations across each sample to account for possible coating heterogeneity. Droplet profiles were recorded immediately after deposition to minimize evaporation effects.

Contact angles were determined using droplet-profile imaging combined with geometric analysis of the liquid–solid–air interface. For each coating condition, multiple droplet measurements were collected and averaged to obtain representative values. Standard deviations were computed to assess variability associated with coating thickness non-uniformity and surface roughness variations introduced during manual deposition. Control measurements were performed on uncoated PET substrates to establish baseline wettability values for comparison.

Table S1. Static water contact angle measurements for PET substrates before and after hydrophobin coating.

Sample	Contact Angle (°) Mean ± SD
Blank PET	60.33 ± 4.62
Lion’s Mane coating	73.67 ± 4.16
Oyster coating	73.00 ± 4.58
Turkey Tail coating	71.33 ± 8.02

Contact-angle measurements were obtained using a profilometer-based droplet profile analysis method, which reduces optical distortion effects associated with off-axis imaging. Reported values represent representative measurements, with summary statistics provided for each condition.

S5. Tribological Measurement System and Calibration

Tribological measurements were performed using a custom-built linear sliding tribometer constructed from modular aluminum 80/20 structural framing components and driven by the motion-control electronics of a modified Prusa 3D-printer controller, which provided repeatable stepper-motor-controlled linear translation. The apparatus was configured to generate controlled horizontal sliding motion while simultaneously recording friction force using an in-line load-measurement system.

S5.1 Mechanical Construction

The tribometer frame was assembled using 80/20 T-slot aluminum profiles to create a rigid base supporting a horizontal sliding carriage. Linear translation was driven through the printer’s stepper-motor system, producing a controlled displacement of approximately 0.30 m (≈1 ft) over 20 s, corresponding to a constant sliding velocity of approximately 15 mm s^{-1}.

The sliding specimen was mounted on a stainless-steel sled positioned on the stationary test surface. A gravity-defined normal load of 1 lbf was applied to the contact interface

by adjusting the sled mass. Because the applied load was fixed at 1 lbf, the measured friction force numerically corresponded directly to the coefficient of friction, simplifying interpretation of the recorded data.

S5.2 Force Measurement Configuration

Friction force was measured using a uniaxial load sensor anchored to the vertical gantry screw assembly of the frame. A Kevlar tension wire connected the moving sled to the load sensor. To maintain horizontal pulling direction, the wire passed over a low-friction nylon pulley connected on a ball bearing, which redirected the force vector by 90° while preserving force magnitude. This configuration ensured that the measured tensile force corresponded directly to horizontal sliding resistance at the contact interface.

The load-cell signal was recorded continuously during each sliding cycle, providing time-resolved force traces from which static and dynamic friction behavior were determined.

S5.3 Calibration and Measurement Consistency

Prior to testing, the load sensor was calibrated using known reference weights applied along the measurement axis to verify linearity and measurement repeatability. Sliding velocity, displacement distance, and normal load were maintained constant across all experiments. All experiments were conducted under ambient laboratory environmental conditions (approximately 22–25 °C and 30–50 % relative humidity).

The tribological measurements presented in the manuscript are intended as representative illustrative observations, demonstrating the qualitative influence of hydrophobin coatings on hydrated interfacial friction rather than serving as standardized tribological certification tests.

S5.4 Tribological Testing Procedure

Tribological measurements were conducted using a sled-type linear friction testing configuration designed to evaluate interfacial sliding behavior under controlled loading conditions. Test specimens were mounted on a weighted sliding carriage positioned on the stationary substrate surface, forming the tribological contact pair used for friction evaluation. Each condition was tested in three independent sliding trials.

During each experiment, the sled assembly was translated horizontally across the test surface at constant velocity using the motorized linear motion system described in Section S5.1-S5.2. The applied normal load was defined by the mass of the sled assembly and maintained constant throughout each test. The pulling force required to initiate and sustain sliding motion was measured using an in-line uniaxial force sensor connected to the sled via a Kevlar tension wire routed over a low-friction pulley to ensure horizontal loading alignment.

Friction force was recorded continuously as a function of time during each sliding cycle. The maximum force recorded at the onset of motion was used to estimate the static friction condition, while the steady-state sliding force obtained after motion stabilization was used to determine the dynamic friction condition. Because the applied normal load was fixed at approximately 1 lbf, the measured friction force numerically corresponded to the coefficient of friction, simplifying interpretation of the experimental results.

For each material or coating condition, 3 sliding trials were conducted under identical operating parameters, and the resulting force traces were averaged to obtain representative friction behavior. All tests were performed under hydrated interfacial conditions using deionized water at ambient laboratory environmental conditions (approximately 22–25 °C and 30–50 % relative humidity).

The tribological experiments included in this study are intended to provide representative illustrative observations of hydrophobin-mediated friction modification rather than standardized tribological certification measurements.

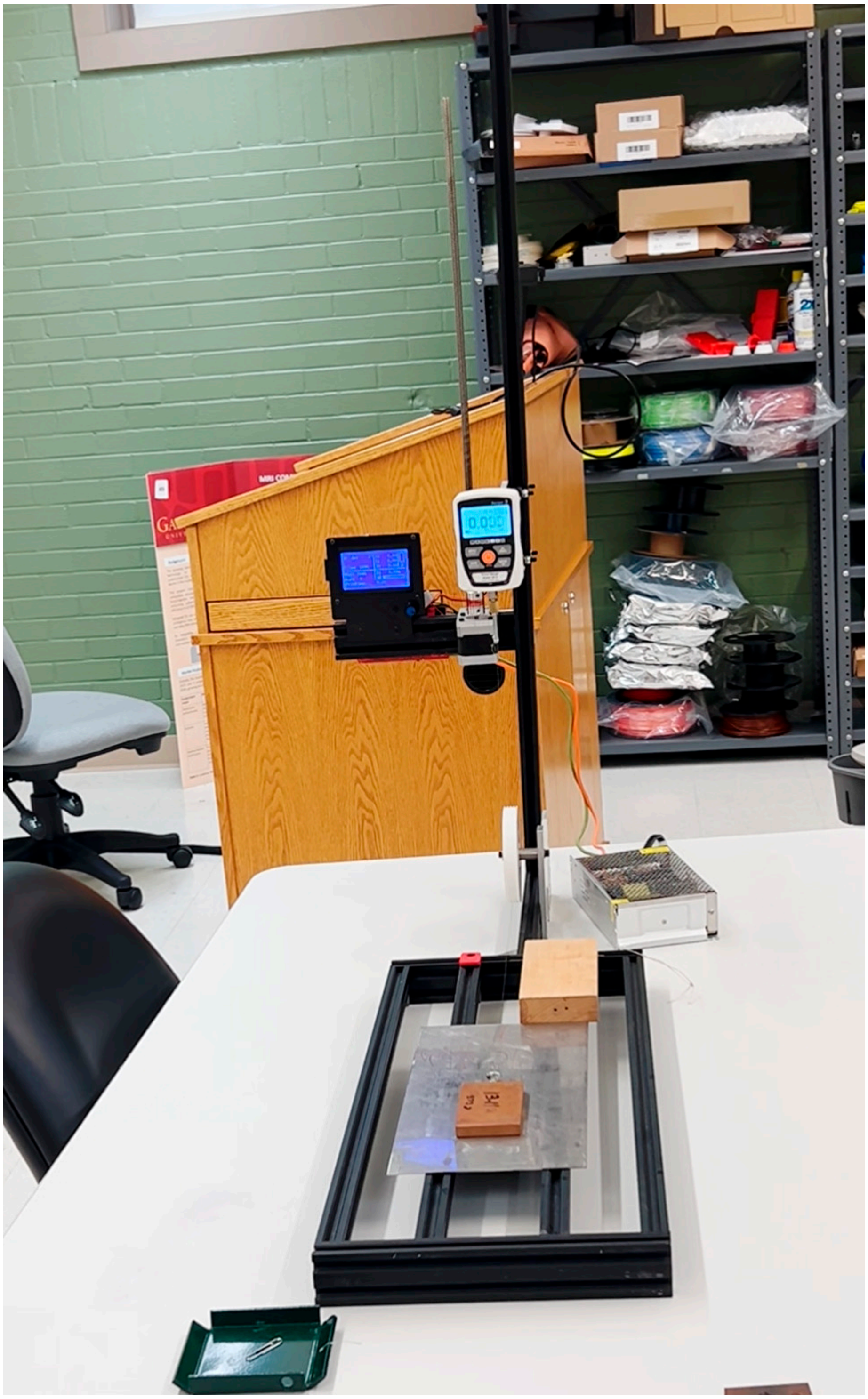

Figure S3. The Figure above shows the friction tester that was used for this experiment.
